# A Complete System of Nursing

**Published:** 1918-02-16

**Authors:** 


### A COMPLETE SYSTEM OF NURSING.

### Reviewed by a Physician and a Trained Nurse.

### THE PHYSICIAN'S VIEW.

The name of the late Miss Honnor Morten will be gratefully recalled by the title of this book. In its present form, the work, it is true, differs widely from the original enterprise, and indeed every page of it has been rewritten for the present issue. None the less, there is due a tribute of appreciation to the lady who primarily undertook the production of a work which sought to bring within the compass of a single volume a scheme of systematic instruction in the art and calling of the trained nurse. Time and circumstance have necessitated change and development, but this is in the nature of things, and in no measure minimises our recognition of the courage which laid the foundations that have made the newer building possible. In appreciating the present volume we desire to remember also the earlier labours out of which it has grown and to recall in a brief word the services Miss Honnor Morten rendered in her day and generation to the profession which she loved so well.

As we turn the pages of Miss Ashdown's volume* we cannot fail to be impressed with the ambitious character of the scheme. Here is an attempt to include the teaching of the whole handicraft of the trained nurse both in its application to medical and surgical cases generally and to the special departments of professional practice, such, for example, as the nursing of mental, ophthalmic, gynæcological, and obstetric cases. Nor does the scheme terminate here. It includes also the practice of massage and of electricity, instruction in sick-room cookery and in the use of drugs and antidotes, and it does not forget a general scheme of advice and exhortation such as is appropriate and even necessary to those who enter on a calling which involves high responsibilities and claims, therefore, an undeviating loyalty and a sincere self-discipline. In this last-mentioned aspect of her subject Miss Ashdown cultivates a lofty ideal, and she associates this culture with a strong common sense born of experience and of good judgment. So far as written advice and exhortation may go the probationer will find in this book excellent counsel relative to the work she has undertaken, as well as shrewd warning against the risks which may attend it. No one with experience will judge either of these directions to be superfluous. The position of the nurse considered as a problem in ethics is far from being a simple one. She has duties and responsibilities to the patients placed under her care, and duties and responsibilities to those to whose direction she is subordinate. It is idle to pretend that she is not sometimes tempted to assume a position of authority to which she has no real claim. Nor when she yields to this is the fault entirely her own. Friends and relatives of patients, naturally anxious and distressed, press for opinions and advice which are quite outside the nurse's functions, and thus force a false position on her. There are, therefore, circumstances in which a firm adherence to subordinate duty and loyalty to superior officers are not always easy, and nothing but effective discipline and the recognition of high principle can prepare the nurse for the straight and steady course. The full training needed to meet such events can only be secured in the atmosphere of the training-school and by actual contact with the practice of those who both know their duty and act on it. But something can be effected by precept, and in Miss Ashdown's book the precept is set forth clearly, firmly, and with all good will.

If the spirit of the training-school is a necessity for the nurse's ethical training, not less essential is the clinical practice of the school to her personal efficiency. For, leaving on one side for the moment its moral bonds and values, nursing is essentially an art or handicraft, and no more than cookery or joinery or bootmaking can it be taught by printed pages. It is in the ward and at the bedside that the nurse has to learn her duty and work. But a book may do much both as a preparation for ward work and also as a reminder of what has been taught in oral and practical instruction. It preserves this teaching in a permanent form, and so may be made to serve as a preparation for examination and as a convenient manual of reference. In these respects it may be fairly stated that Miss Ashdown's book is well adjusted to its ends. There is no failure to recognise the need for lucid and detailed description of the duties which have actually to be carried out by the nurse. Whether it is a question of making a poultice or preparing a patient for an abdominal operation, all is given in an exact, inclusive, and precise fashion. The business is a strictly practical one, and this is recognised in a truly effective fashion. This note prevails throughout, and thus we have no hesitation in saying that the manual is one eminently suited for the probationer, and even the more experienced nurse will find in it hints and suggestions of high practical value.

The one criticism which we would make on the book is one which is almost inevitable when an ambitious enterprise has to be accommodated in a limited space; it is, that concentration in some directions is apt to be excessive. Thus, to take a single example, we should say that the descriptions of the tests for albuminuria and for glycosuria are insufficient to fit the nurse for a piece of work which, under adequate supervision, she may well be expected to perform. On the other hand, a good deal of the sections dealing with symptoms and treatment —necessarily sketchy and superficial as they must be— might well disappear without any appreciable measure of loss. What real value, for example, can be attached to a three or four line account of the symptoms and treatment of Dercum's disease, of myasthenia gravis, of acute bulbar paralysis, or of fatty degeneration of the myocardium? Such limited information is of no practical service, and a parade of it is apt to lead its possessor into dogmatic utterances which have the sound of knowledge but not its substance. Of the rest of the book, as we have already indicated, we wish to speak with appreciation and good will. The work has been done carefully and with thoroughness, and it may be cordially commended to the constituency which it aspires to serve.

### WHAT THE TRAINED NURSE THINKS.

The nursing profession will gladly welcome "A Complete System of Nursing," whose author has done everything to justify the title of this excellent text-book for nurses. The most noticeable feature of this book is that it has obviously been written by a nurse knowing the needs of the sick and the needs of nurses, and for either

* *A Complete System of Nursing.* By A. Millicent Ashdown. (London and Toronto: J. M. Dent and Sons, Ltd. 1917. Pp. 746 + xv. Price 10s. 6d. net.)

